# BOLD Specificity and Dynamics Evaluated in Humans at 7 T: Comparing Gradient-Echo and Spin-Echo Hemodynamic Responses

**DOI:** 10.1371/journal.pone.0054560

**Published:** 2013-01-15

**Authors:** Jeroen C. W. Siero, Nick F. Ramsey, Hans Hoogduin, Dennis W. J. Klomp, Peter R. Luijten, Natalia Petridou

**Affiliations:** 1 Rudolf Magnus Institute, Department of Neurosurgery and Neurology, University Medical Center Utrecht, Utrecht, The Netherlands; 2 Department of Radiology, University Medical Center Utrecht, Utrecht, The Netherlands; University of Minnesota, United States of America

## Abstract

High-field gradient-echo (GE) BOLD fMRI enables very high resolution imaging, and has great potential for detailed investigations of brain function. However, as spatial resolution increases, confounds due to signal from non-capillary vessels increasingly impact the fidelity of GE BOLD fMRI signals. Here we report on an assessment of the microvascular weighting of the GE BOLD response across the cortical depth in human cortex using spin-echo fMRI which is thought to be dominated by microvasculature (albeit not completely). BOLD responses were measured with a hemodynamic impulse response (HRF) obtained from the spin-echo (SE) and gradient-echo (GE) BOLD contrast using very short stimuli (0.25 s) and a fast event-related functional paradigm. We show that the onset (∼1.25 s) and the rising slope of the GE and SE HRFs are strikingly similar for voxels in deep gray matter presumably containing the most metabolically demanding neurons (layers III–IV). This finding provides a strong indication that the onset of the GE HRF in deep gray matter is predominantly associated with microvasculature.

## Introduction

In vivo exploration of brain function has seen impressive progress in the last decade and moves towards resolving increasingly finer details. Especially functional magnetic resonance imaging (fMRI) at high field strength shows promises of investigating functionally specialized regions, even down to the laminar and cortical column scale. The experimental requirements for such in-depth investigations are methods with high sensitivity but most of all methods with high specificity. Ideally, these methods should be specific to the microvasculature that directly serves active neuronal sites. For neuroscientists, the fMRI method of choice with superior sensitivity is gradient-echo (GE) BOLD imaging. Though GE BOLD has opened up the possibility to visualize the columnar and laminar functional organization in the cortex [1]–[4], it can suffer from non-specific contributions of several vessel sizes.

Recently, using GE BOLD imaging we showed that the hemodynamic response shape can be characterized across the cortical depth in human primary cortices [5]. One of the prime findings was that the fastest onset times of the response are found in the deep gray matter, signifying that GE BOLD can resolve the capillary-dense deep gray matter. In order to independently confirm that GE onset times can specifically mark the timing of events in the capillary-bed a comparison is required with a response solely from the microvasculature. This evaluation should reveal the different vascular contributions to GE BOLD fMRI at every cortical depth. However, no in-vivo method exists that meets this requirement. The SE BOLD contrast constitutes the next-best reference since, although contributions from larger vessels can still be present, it is the technique that is the most dominated by capillary signals [6]–[8]. The magnitude of contributions from larger vessels depends on the finite EPI readout-length resulting in T2* contamination when employing echo-planar-imaging (EPI) for acquiring SE BOLD data [9]. At high field strength (≥7 T), simulations and experiments reveal reduced contributions from postcapillary and surface pial vessels, favoring the microvascular weighting of SE BOLD [7], [8], [10]–[15]. Importantly, a remaining challenge in measuring a microvascular-weighted hemodynamic response function (HRF) with high spatiotemporal fidelity is the intrinsically low sensitivity of the SE BOLD contrast [10].

Here we compare the GE BOLD HRF obtained across cortical depth with a SE BOLD HRF measured at 7T. This study is the first to estimate the SE BOLD HRF waveform where we overcome the low sensitivity of the SE BOLD contrast by employing a dedicated multichannel surface coil [16]. With this setup, we acquired SE and GE BOLD event-related data with high spatial and temporal resolution and very short visual stimuli (250 ms). Given the low SNR in SE-EPI and the need for SNR for estimating the HRF, resolution was traded for minimal T2* weighting in the EPI readout and sufficient signal-to-noise ratio. This approach allowed us to obtain the HRF approximating the impulse response of the vasculature upon neuronal activation. The neurovascular dynamics can then be analyzed as a linear system. Upon neuronal activation, blood flow is increased in the capillary bed in the deeper cortical layers directly serving the active neuronal sites [17], [18]. Next, blood flows into the postcapillary venules and intracortical veins ascending to the cortical surface and is finally drained by the larger pial veins at the cortical surface [17]–[19]. Considering these blood flow dynamics upon neuronal activation we anticipate that the shape of a microvascular dominated HRF should show a short onset time and a narrow response as compared to an HRF containing contributions from blood draining into the larger postcapillary vasculature and pial vessels.

Comparing the SE HRF with the GE HRF enabled us to estimate the different vascular contributions to the GE HRF across the cortical depth. Here, we expect that similarities between the SE and GE HRF represent an estimate of the microvascular contributions to the GE HRF (acknowledging some larger vessel contributions). The cortical depth was divided in three sections; 0–1 mm, 1–2 mm and 2–3 mm. From anatomy studies [20], [21], we anticipate in the 1–2 mm and 2–3 mm sections (hereafter defined as ‘deep gray matter’ region) a high capillary density together with a sparse but well-structured postcapillary vasculature. For the 0–1 mm section (defined as the ‘surface gray matter’) we expect a more dominant contribution of the macrovasculature. The SE and GE hemodynamic response evolution was compared with respect to onset time, time-to-peak (TTP), full-width-at-half-maximum (FWHM) and maximum percent signal change (PSC). Results reveal that the onset time and rising slope are almost identical for the GE and SE HRF, specifically at a cortical depth >1 mm. These findings indicate that high-resolution GE-BOLD is weighted towards the microvasculature in the early phase of the response in deep gray matter.

## Materials and Methods

Eight healthy subjects participated in the study after giving written informed consent in accordance to the Institutional Review Board of the Utrecht University Medical Center. FMRI data of the primary visual cortex were acquired using a gradient-echo (GE) and spin-echo (SE) scan employing an identical fast event-related fMRI (ER-fMRI) paradigm using short 250 ms visual stimuli. The HRF was estimated for significantly active voxels in primary visual cortex (V1) in both scans. For the GE scan the estimated HRFs were categorized according to their cortical depth and compared with the SE BOLD HRF. The methods are described in detail below.

### Data acquisition

The subjects were scanned at a Philips 7 T MR system with a dedicated 16 channel surface coil [16], and a standard birdcage volume transmit coil. GE functional data were obtained using a multislice single-shot GE-EPI acquisition with TR/TE = 880/27 ms, flip angle = 70° (effective flip angle  = 42–50°, after adjusting for the B1 transmit field in the visual cortex [16]), SENSE factor  = 3, bandwidth in the phase encoding direction (BW_phase_) = 29.4 Hz/pixel, an isotropic voxelsize of 1 mm, FOV = 125×121 mm^2^, and 7 slices covering the primary visual area V1. SE functional data were obtained using a multislice single-shot SE-EPI acquisition with TR/TE = 880/50 ms, flip angle = 125°, SENSE factor  = 2, BW_phase_  = 37.0 Hz/pixel (readout length 27 ms), an isotropic voxelsize of 2 mm, FOV = 190×182 mm^2^, and 5 slices covering the same central area as the GE scan. 3^rd^ order image based shimming was performed on the FOV of the functional scans after brain extraction (BET [22]), using in-house developed software in IDL (v6.3 RSI, Boulder, CO, USA). A high resolution whole brain T_2_*-weighted scan was acquired as an anatomical reference, and to visualize the large draining veins, with the following parameters: 3D multishot GE-EPI, TR/TE = 90.8/27 ms, FA = 20°, 2 averages, SENSE factor = 2 in the phase-encoding direction, BW_phase_  = 34.2 Hz/pixel, an isotropic voxelsize of 0.5 mm and FOV = 188×188×30 mm^3^. An EPI read out was used for the anatomical scan in order to have similar geometric distortions as in the functional images [23]. Cardiac and respiratory rate data were recorded during all scans for subsequent correction of physiological noise.

### Functional Paradigm

Functional data for both GE and SE acquisitions were obtained employing an identical fast ER-fMRI paradigm which consisted of four parts; i) 31 s baseline period, ii) 464 s event-related (ER) part, iii) 31 s baseline period and, iv) 79 s block design (localizer) part with off/on periods of 15.8/15.8 s (uniform gray screen/8 Hz reversing checkerboard). The event train for the ER part was generated with interstimulus intervals (ISI) taken from an exponential distribution with mean/min ISI  = 8.2/2.9 s [24]. In total 54 stimuli were presented with a stimulus duration of 250 ms (two 125 ms opposing checkerboard frames). The stimulus onset was uniformly jittered relative to the TR, yielding a sub-TR temporal resolution of 220 ms. Short stimuli together with a minimal ISI of 2.9 s will yield a narrow HRF with minimal hemodynamic nonlinearities [15], [25], [26]. All conditions included a central red fixation point.

### Data processing and analysis

All functional scans were corrected for motion and linear drift, and then corrected for cardiac and respiratory fluctuations using RETROICOR [27]. The localizer part was processed using FEAT including high pass filtering (cut-off at 1/31.6 Hz.), Z threshold  = 3, slice timing correction and no spatial smoothing (FSL, FMRIB Software Library, Oxford). The largest significant cluster in V1 (cluster P threshold  = 0.05, corrected for multiple comparisons) was selected and used as a region of interest for the ER-fMRI analysis. Large draining veins (and extravascular space, specified as a 1 mm radius around the vein) were identified based on their low intensity on the high resolution T2*-weighted scan, and excluded from the GE functional data. Signals from these vessels are the least specific in terms of localizing active neuronal tissue as they drain from multiple downstream sites [28]. Estimation of the HRF was performed by means of conjugate gradients for deconvolution [29] after normalizing the BOLD time courses by the baseline signal (mean of the two baseline periods) and 8-fold Fourier interpolation (yielding an effective sampling time of 880/8 = 110 ms). This level of interpolation was chosen for the purpose of slice-timing correction. Slice-timing correction was performed simultaneously with the HRF estimation by shifting the stimuli model for each slice according to the slice acquisition time.

#### Cortical depth analysis and GE vs. SE BOLD HRF comparison

For the GE data, the cortical depth in V1 was estimated for each HRF where the cortical surface was delineated manually in 3D on the high resolution, 0.5 mm isotropic, T_2_*-weighted anatomical image after coregistration to the GE data. The shortest distance to the cortical surface was computed for each active voxel from the GE functional scan in the 0.5 mm grid where each voxel was represented by 8 smaller voxels due to the two-fold interpolation in three dimensions. The shortest distance was defined as the Euclidean distance from the centre of each 8-voxel group to the cortical surface delineation thus giving the distance to the voxel originally sampled. The cortical depth for each voxel was then defined as the computed shortest distance and binned in three sections; 0–1 mm, 1–2 mm and 2–3 mm (3 mm is approximately the cortical thickness [30], [31]). The TTP, FWHM, PSC, and onset time was computed from the average HRF for each section. The onset time was defined by fitting a line to the slope between 20% and 80% of the peak of the HRF and computing the intercept with the baseline [18]. Note that voxels including or adjacent to large vessels were excluded from this analysis. For the SE data, the TTP, FWHM, PSC, and onset time was computed for the average HRF of all active voxels in V1. Because different contrast mechanisms produce the GE and SE responses we can expect amplitude differences in terms of maximum percent signal change. Therefore, a more quantitative measure of the vascular contributions to the GE HRF was obtained by dividing the GE and SE HRFs by the used echo times (27 ms and 50 ms respectively) which yields the contrast relaxation rates ΔR2*(t) and ΔR2(t) respectively [32]. Furthermore, the ratio of ΔR2* to ΔR2 relates to the vessel size where a larger ratio means larger vessels are probed [33]. Next, the computed ratio of ΔR2*(t) and ΔR2(t) was examined over time, in two parts; the earlier part of the HRF, defined as the 20% peak value of the rising slope up to the TTP of the SE HR, and the later part of the HRF, defined as the period from the TTP to the 20% peak value of the falling slope. The average ΔR2*(t)/ΔR2(t) ratio was also computed across subjects as a function of SE TTP time (i.e. fraction of SE TTP), hence all the individual ratio profiles could be aligned with respect to the SE TTP time point.

## Results

### Spatial maps and time courses of the GE and SE HRFs

**Figure 1:**
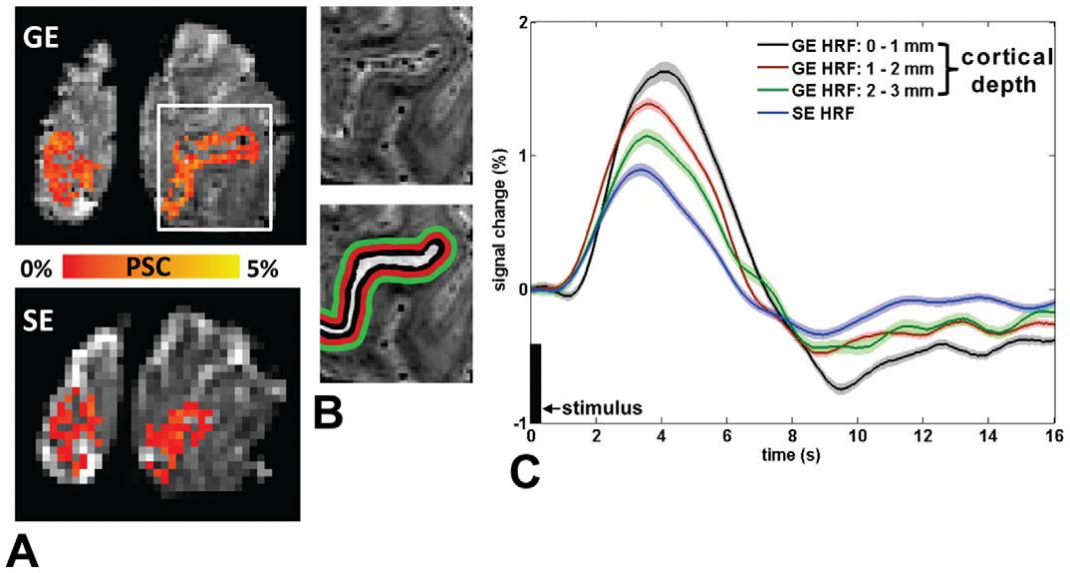
Spatial activation maps and time courses of the GE and SE HRF upon short visual stimulation. A) Percent signal change (PSC) map of the visual cortex V1 for the GE (top) and SE (bottom) HRF data with isotropic voxel sizes of 1 mm and 2 mm, respectively. As expected the SE contrast shows reduced sensitivity. B) Closer examination of the cortical surface and pial vasculature on the 0.5 mm T2*-weighted anatomical scan. The cortical surface (white) was manually delineated in 3D and from this surface the cortical depth profiles were computed: black; 0–1 mm, red; 1–2 mm, and green; 2–3 mm. The high-resolution T2*-weighted scan was also used to identify the larger pial draining veins, which were excluded from the GE BOLD analysis. C) GE HRFs across cortical depth (0–1, 1–2, and 2–3 mm) and the SE HRF for a representative subject (subject 4 as indicated in Table 1). As the GE and SE BOLD do not measure from the exact same vasculature there should be no exact spatial match in their activation patterns from the functional localizer. We therefore focused on the temporal evolution of the estimated HRFs, with the following parameters of interest: onset time, time-to-peak (TTP), full-width-at-half-maximum (FWHM), and maximum percent signal change (PSC). Onset times of the SE and GE HRF in deep gray matter (>1 mm cortical depth) are very comparable while the FWHM and TTP are increased for the GE HRF for all cortical depths indicating that the earlier part of deep gray matter GE HRF is weighted toward microvascular dynamics. Shaded areas denote the standard error of the mean (SEM). The black bar indicates the stimulus onset and duration (250 ms).

**Figure 2:**
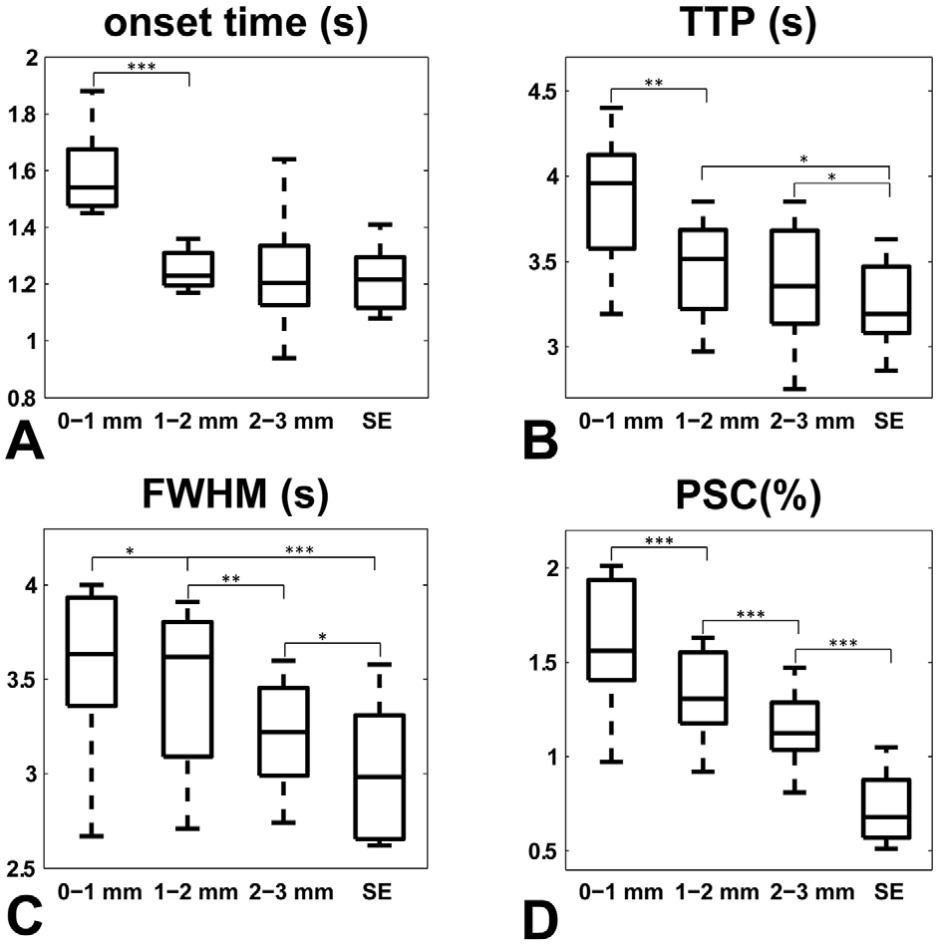
Box plot of the temporal HRF properties of the GE HRF across the cortical depth and SE HRF across all subjects. A) Onset time (s), B) TTP (s) C) FWHM (s), and D) PSC (%). Comparing the SE HRF with surface gray matter GE HRF (0–1 mm cortical depth) shows that all temporal properties are significantly increased (P<0.01, Wilcoxon signed–rank test). This indicates that the surface gray matter GE contrast probes a different part of the vascular system upon activation than the SE contrast. Surface gray matter in V1 contains mainly postcapillary veins (diameters ∼70 μm) and much less capillaries (diameters ∼5 μm) than deep gray matter regions [20]. Looking at the deep gray matter GE HRFs (>1 mm cortical depth) and the SE HRF we found that the TTP, FWHM and PSC were all significantly increased for the GE HRF. The onset times, however, did not differ significantly. These findings suggest that the earlier part of the deep gray matter GE HRF is in close correspondence to the SE HRF and hence is weighted towards the microvasculature. ^***^ denotes significant difference between the two compartments (Wilcoxon signed–rank test for P<0.01, ** for P<0.05, * for P<0.1).

Significantly activated tissue in the primary visual cortex (V1) was identified for both the GE and SE data with a block-design functional localizer task. For each of the voxels identified as active, the HRF was estimated from the subsequent fast event-related data obtained with 250 ms visual stimuli. Example activation maps for both the GE and SE event-related data are shown in Figure 1A. As expected we observe a reduced sensitivity of the SE data as compared to the GE data by the lower percent signal change (PSC) of the hemodynamic response (Figure 1A, Figure 1C, and Figure 2D). Figure 1B (white ribbon) illustrates, superimposed on the 0.5 mm^3^ resolution T2*-weighted anatomical scan, the delineation of the cortical surface (calcarine sulcus, V1) and the corresponding cortical depth sections; 0–1 mm, 1–2 mm, and 2–3 mm indicated by the black, red and green ribbons respectively.

### Temporal characteristics of the SE HRF and GE HRFs across cortical depth

Responding to the short visual stimulus, the GE HRF initiated within 1.25 s at cortical depths of 2–3 and 1–2 mm (Figure 1C; red and green curves, and Figure 2A). This timing matched that of the SE HRF onset (Figure 1C; blue curve, and Figure 2A), indicating that early hemodynamics probed by GE BOLD are weighted towards the microvasculature at these cortical depths. The GE HRF onset timing of the surface gray matter (Figure 1C; black curve) was delayed by ∼0.35 s as compared to the GE HRF onset from the deep gray matter, i.e. both the 2–3 mm and 1–2 mm sections, and also with respect to the SE HRF onset time (Figure 2A).

As compared to the SE HRF, we find an increase in FWHM and TTP for GE BOLD of 0.55, 0.45, 0.28 s and 0.62, 0.21, 0.12 s respectively for the three different cortical depth sections; 0–1, 1–2, and 2–3 mm (Figure 2B and Figure 2C). The deviation in TTP between the SE HRF and the GE HRF obtained from deep gray matter gives an estimate of the time at which postcapillary contributions that are not measurable in the SE HRF become measurable in the GE HRF. The GE HRF from the 2–3 mm section has a similar onset time to the SE HRF however the TTP shows a slight deviation at trend level; ΔTTP  = 0.12 s (P<0.1). Subtracting the onset time from the TTP value of the GE HRF we can estimate that postcapillary contributions are clearly observable in our GE data at least ∼2 s after onset of the GE HRF in the deep gray matter. For the surface gray matter we find an overall delayed temporal response in terms of onset, FWHM, and TTP as compared to both the SE and the deeper GE HRF.

### Time course of vascular contributions to the GE HRF

**Figure 3:**
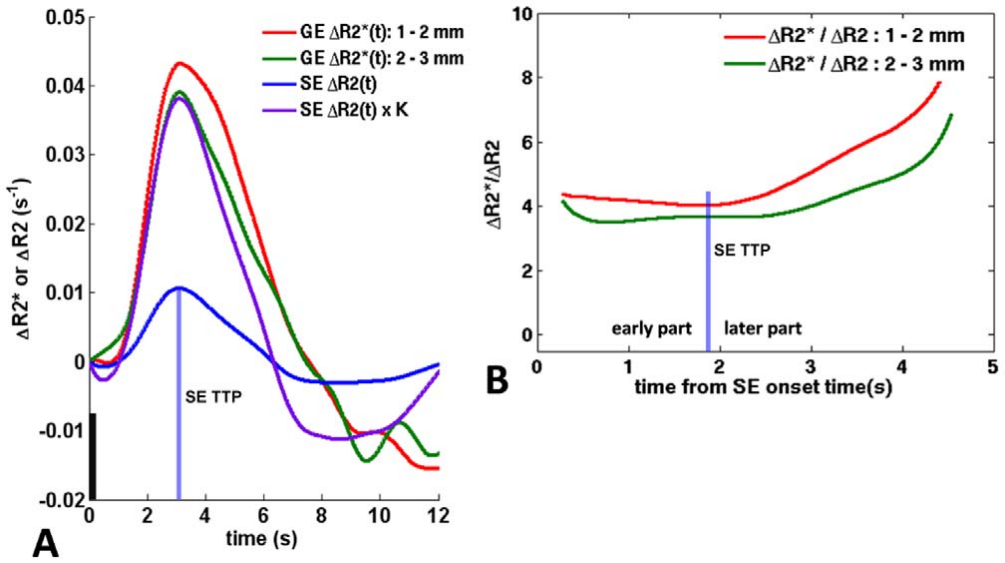
Assessment of the vascular contributions to the GE HRF upon short visual stimulation. A) The resulting GE HRFs and SE HRF for a representative subject (subject 1 as indicated in Table 1) when normalized by the used echo time, yielding ΔR2*(t) and ΔR2(t) [32]. The purple curve shows the scaled ΔR2(t) of the SE HRF by the ratio of ΔR2*(t) to ΔR2(t) (factor K) to account for the different contrast mechanisms. Scaling by this ratio makes it possible to compare directly the SE HRF evolution with the GE HRFs across cortical depth. K is the average ratio of the earlier part of the deep gray matter GE HRF (2–3 mm) (Figure 3B and Figure 4). B) Curves of the ratio of the GE versus SE measure (ΔR2*/ΔR2) across cortical depth (1–2, and 2–3 mm cortical depth) of the same subject. For the deep gray matter, the ratio remains constant up to the TTP (blue bar), and increases in the later part. This indicates microvascular weighting in the early part of the deep gray matter GE HRF. The surface gray matter GE HRF was omitted here because of the large offset in onset time with respect to SE HRF. The black bar in A) shows the stimulus onset and duration (250 ms). The blue bar in both panels marks the TTP of the SE HRF.

**Figure 4:**
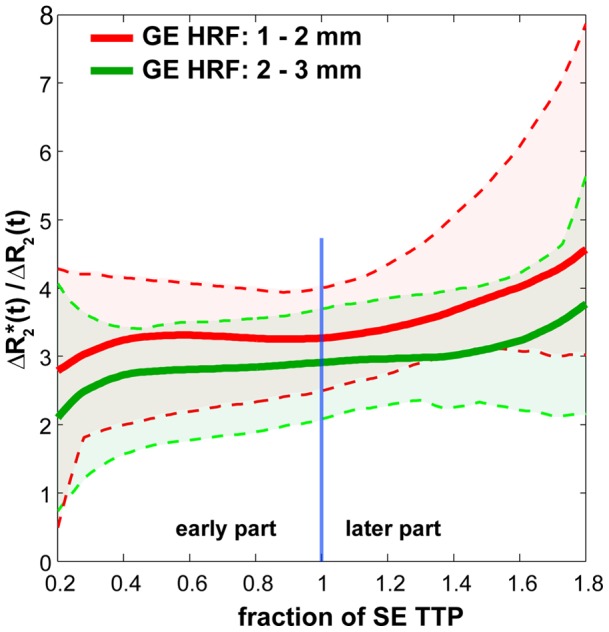
Vascular contributions to the GE BOLD HRF averaged over all subjects. Average ratio curves ΔR2*(t)/ΔR2(t) over all subjects to directly track the SE HRF contribution over time in the deep gray matter GE HRFs (1–2 mm; red, 2–3 mm; green). In deep gray matter, for the period of 40% to 100% of the SE TTP (indicated by the blue bar) ratio curves are relatively constant. This demonstrates that the early part of the GE HRF in the deep gray matter contains very similar contributions as the SE, i.e. weighted towards the microvasculature. From the SE TTP and onwards the ratio deviates considerably from the early part which we hypothesize reflects increasing vascular contributions. The ratio profile for each subject was corrected for the different TTP values between the subjects; the profiles were computed as a function SE TTP time (i.e. fraction of SE TTP), in this way all the individual ratio profiles could be aligned with respect to the SE TTP time point. The dotted lines indicate the minimum and maximum ratio profiles across the subjects.

Figure 3A shows the resulting ΔR2*(t) and ΔR2(t) curves for a representative subject. Figure 3B illustrates how the ΔR2*(t)/ΔR2(t) ratio was computed from the GE and SE HRFs in Figure 3A for the individual subjects. Figure 4 shows the average the ΔR2*(t)/ΔR2(t) ratio across subjects where the individual ratio profiles were shifted to match the individual subject's TTP. The expected ratio for gray matter is on the order of ∼2.6 considering the contrast mechanisms [13]. We find an average ratio of 2.85±0.64 across subjects for the early part of the GE HRF (2–3 mm) (see K values in Table 1), while the ratio increased for the later part and remaining cortical depth sections (Figure 4 and Table 1). We further find that the deep gray matter GE and the SE HRF closely match during most of the rising slope (40%–100% of TTP). This can also be seen by scaling the SE by the ratio, which allows for a direct comparison of amplitude change (Figure 3A, purple curve). We did not consider the surface gray matter in this analysis because it was clearly dominated by the macrovasculature as seen by the overall HRF delay and widening.

**Table 1:** ΔR2*/ΔR2 ratios for all subjects for the early and later part of the GE HRF (2–3 mm and 1–2 mm sections).

	2–3 mm gray matter section		1–2 mm gray matter section	
Subject	ΔR2*/ΔR2 early part (K)	ΔR2*/ΔR2 later part	ΔR2*/ΔR2 early part	ΔR2*/ΔR2 later part
1	3.63	4.42	4.17	4.67
2	2.65	3.05	2.64	3.54
3	3.13	2.73	3.77	3.41
4	2.86	3.77	3.31	3.62
5	2.08	2.93	2.24	3.00
6	1.79	2.46	2.25	3.01
7	3.35	4.60	3.89	5.41
8	3.27	4.08	3.33	3.75
mean ± std:	2.85±0.64*	3.51±0.82	3.20±0.75*	3.80±0.83

Normalizing the SE and GE HRFs across cortical depth and by the used echo times, yielding ΔR2*(t) and ΔR2(t) respectively, makes it possible to compare two contrasts in more detail [32]. The ratio of ΔR2* to ΔR2 is an indication of the measured vessel sizes [33]. Larger ratio means larger vessels are probed. The ratio was computed for the GE HRF at 2–3, i.e. the K value in Figure 3A, and 1–2 mm cortical depth sections using the SE HRF.^*^ denotes a significant difference with column to the right, one-sided paired Student's t-test for P<0.01.

## Discussion

GE BOLD fMRI at high fields shows promises to investigate the cortical columnar and laminar functional organization. However, several important issues concerning the fidelity of the signal need to be resolved. Here we address the different vascular contributions to the GE BOLD signal, an issue that has an impact on the spatial accuracy of the source of the BOLD signal as it relates to neuronal activity. Our results indicate that the early phase (onset and rising slope) of the GE HRF in deep gray matter (>1 mm depth) is weighted toward the microvasculature, while macrovascular dynamics become observable at the later phase of the response and are predominant in the surface gray matter. By comparing the GE BOLD hemodynamic impulse response to the SE BOLD hemodynamic impulse response we find that the onset and rising slope of the GE and SE HRF are strikingly similar. This was specifically observed at a cortical depth greater than 1 mm where presumably the most metabolically demanding neurons are located (cortical layers III – IV).

Results of the HRF properties for surface gray matter (<1 mm depth) yielded a delayed temporal response in terms of onset, FWHM, and TTP as compared to both the SE and the deep gray matter GE HRF (Figure 2). This signifies dominating macrovascular contributions (downstream draining) and potential pial surface signal (traversing surface vessels) contributions. Extravascular signal contributions from the pial surface should not affect the HRF onset time in deeper gray matter but only the TTP and FWHM parameters due to the time it takes blood from the gray matter to enter the pial vasculature. Deeper in the cortex, microvascular weighted signal changes are clearly detectable in the onset and rising slope of the response. Here, the deeper gray matter was examined in two sections, spanning depths of 1–2 mm and 2–3 mm from the cortical surface. We find strikingly similar HRF onset times in the two deep gray matter sections, namely 1.24 and 1.25 s which matches the onset of the SE HRF, suggesting a distinct local vascular control in the cortical areas corresponding to these gray matter sections. This indicates that the local capillary vessels react at the same moment to the stimulus as opposed to passive downstream draining. This local response is thought to serve the metabolic demand at these cortical depths and is directly regulated by the active neurons and supporting glial cells [34]–[36]. This implies that GE BOLD can be weighted towards the microvasculature but that it may also be able to resolve individual hemodynamic regulation patterns within gray matter. However more studies are required to thoroughly investigate this effect.

### Vascular dynamics of the deep gray matter GE HRF

Postcapillary contributions to the GE HRF from deep gray matter (>1 mm) are identified at later time points in the waveform. Blood, after residing for some time in the capillaries (velocity 0.7 mm/s for rats [37]), is collected by a well-structured network of venules and intracortical veins [20]. Blood collected in this postcapillary network is an accumulation of blood with different transit times resulting from the different paths across the vascular compartments. This can result in a peak shift and widening of the overall measured hemodynamic response, which would lengthen the response width (FWHM) and TTP of the GE BOLD contrast from the different cortical depth sections as compared to the SE BOLD response (Figure 2B and Figure 2C). We find an increase in TTP and FWHM of the GE HRF of ∼0.2 s and ∼0.4 s respectively (averaged difference of the two deep gray matter sections), suggesting that later time points of the GE HRF contain increased postcapillary contributions as compared to the SE HRF. The increased peak time marks the time-point of these postcapillary contributions which we estimated at ∼2 s after onset (Figure 2 and Figure 3B). One needs to keep in mind that earlier time points of both the SE and GE HRF may also contain postcapillary contributions though they may be outweighed by the bulk of microvascular signal changes. The TTP and FWHM was prolonged for the 1–2 mm section (middle gray matter section) as compared to the 2–3 mm (deepest gray matter) section which may be anticipated if blood from deeper gray matter is draining in the larger vasculature in the middle section. However, these differences did not reach statistical significance perhaps due to the dense capillary-bed through these depths in V1 which dominated the responses. Additionally, the blood volume of venules and intracortical veins may have not been sufficient to create a significant difference in these cortical regions. Notably, in our previous study using GE BOLD, employing a larger voxelsize (1.5 mm isotropic), significant differences were found for these parameters at these cortical depths [5]. An explanation could be that the larger voxel size increases the partial volume effects in the data which could cause some contribution from the surface gray matter to the middle gray matter section, increasing the TTP and FWHM. Similarly, at the resolution used here (1 mm isotropic) partial volume effects are still likely present across the different vascular layers of gray matter, and may still contribute to the HRF properties in the three gray matter sections. Imaging at an even higher spatial resolution (and at high temporal resolution) may allow further discrimination of the micro- and macrovascular contributions to the temporal evolution of the vascular response. Further, the three cortical depth sections may contain a variable mixture of vascular (and neuronal) layers due to potential variations in cortical thickness. Such variability may blur a local vascular response arising in the cortical layers presumably contained at a given cortical depth. The similarity in onset times in the deep gray matter sections suggests that these effects, if any, are likely detected at later time points. It is therefore possible that the differences in TTP and FWHM between the deeper gray matter sections may be further reduced if variations in cortical thickness are taken into account.

When we consider the ΔR2* to ΔR2 ratio profile of the two deep gray matter sections (Figure 4), we see that the ratio increases for the later part of the HRF (see also Table 1). This indicates that larger vasculature is probed at later time points with accumulating contribution over time. The relatively flat ratio in the early part indicates a similar evolution between SE and GE HRFs (Figure 3B and Figure 4). There is however a small drop in the ΔR2*/ΔR2 ratio in the beginning of curve in Figure 4 which is probably due to the fact that the deep gray matter GE and SE HRF did not have the exact same onset time for all subjects (also visible in Figure 2A in the onset time difference between the 2–3 mm GE and the SE HRF). Although not significant, the SE HRF onset time was on average a bit shorter than the GE HRF in deep gray matter (Figure 2A). Computing the ΔR2*/ΔR2 ratio can then lead for some subjects to very low ΔR2*/ΔR2 values in early time points of the ratio profile. A recent study at 7 T suggested that GE BOLD fMRI using a TE that is shorter than the T2* of gray matter (∼27 ms [8] as used here) may potentially improve the sensitivity to the microvasculature in the middle layers [38], which may improve the onset time estimation of the deep gray matter GE HRFs. It is possible that the small differences between onset times for the deep gray matter GE and SE HRFs may be reduced at shorter TEs.

### Partial volume effects of the SE BOLD data

Additionally, we observed an overall reduction in the ΔR2*/ΔR2 ratio for the deepest gray matter GE HRF as compared to the middle gray matter GE HRF. It is likely that a large part of the capillary-bed resides in the middle cortical depth section. Therefore the SE HRF, governed by the ΔR2 over time from a 2 mm voxel, is more weighted to the capillary and likely postcapillary signal changes in the middle cortical depth section. As at this cortical depth the gray matter may contain a higher capillary density, and hence more blood volume, this will increase the ΔR2. Consequently this reduces the ΔR2*/ΔR2 ratio for the deepest gray matter GE HRF. Besides a partial volume effect across multiple vascular layers there is a possibility of gray matter partial volume with white matter and cerebrospinal fluid, which can affect the CNR and shape of the SE HRF. In the case of partial volume with white matter, the CNR of the SE HRF would be reduced but the HRF shape would not substantially alter in terms of onset time, TTP, and FWHM. However, partial volume with CSF could result in macrovascular contributions, thus reducing the microvascular specificity of the SE HRF in voxels containing pial veins surrounded by CSF. Due to the much higher diffusion constant of water protons in CSF as compared to gray matter (almost a factor 3, [39]), the SE BOLD contrast can potentially become sensitive to extravascular BOLD effects of pial veins in CSF. The high diffusion constant can lead to a dynamic averaging component of these extravascular BOLD effects which can then contribute to the SE BOLD signal. Ideally one would match the SE data voxel size to the GE data voxel size, i.e. 1 mm isotropic, in order to reduce partial volume effects and to obtain a more optimal voxel selection and comparison across the cortical depth. This, however, comes at the price of a substantial reduction in SNR. Only with the use of a dedicated surface coil we could achieve the current SE HRF estimation using a 250 ms stimulus duration, sub-second TR and sufficient coverage of V1, all prerequisites for adequate measurement of the temporal dynamics of the SE HRF. Also, smaller voxels will result, with the constraint of sub-second TR, in much longer EPI readouts and hence more ΔR2* contamination of the SE HRF. We note that our employed SE fMRI acquisition does not yield a pure ΔR2 contrast either, but contains some ΔR2* contamination due to the finite length of the EPI readout (27 ms). At worst this may result in pial vasculature signal contributions entering into the SE HRF. Both the potential contributions from pial veins in the CSF and ΔR2* contamination can impede the microvascular specificity in SE BOLD. We anticipate that these contributions are minimal as both would substantially broaden the SE HRF as compared to the deepest gray matter GE HRF which is not what is observed. The SE HRF was found to have a ∼0.3 s more narrow response as compared to the deepest gray matter GE HRF (Figure 2C).

### Vascular dynamics of the surface gray matter GE HRF

For the surface gray matter (cortical depth <1 mm), as blood is entering and pooling in the larger vasculature, we find large increases in TTP and FWHM (Figure 2), but also a significant delay in onset time of ∼0.35 s as we have previously reported [5]. However, this delay is much less than expected from the transit time of blood across the cortical vasculature as measured in rats (∼1 s) [37]. One possible explanation is that postcapillary contributions were detectable earlier, for example signals from blood arriving at the surface gray matter via the intracortical veins from the middle cortical depth [19]. It is also possible that blood transits faster through the cortical vasculature in humans or that a locally initiated vascular response is detectable in the surface gray matter. As it is difficult to translate findings between species due to differences in vascular biology and experimental setup (e.g. anesthesia), further in-depth investigation on cortical blood transit in humans seems warranted.

### Linearity of the BOLD signal to neuronal activity

The hemodynamic response to a neuronal event is a composite of multiple stages in the vascular response tree which consists of changes in blood flow, volume and oxygenation for the different vessel groups (capillaries, venules, intracortical veins). The hemodynamic changes may be induced actively (e.g. vasodilatory signals) or passively (e.g. blood pooling), with variable contributions across cortical lamina that match the vascular organization across cortical depth. SE BOLD can allow insights to the microvasculature but its practical use is limited because of SAR restrictions and because its sensitivity is low even at high fields [7] (see also Figure 2D). The work here investigates the microvascular weighted component of the GE curve, i.e. the vascular response element most directly linked to a neuronal event. Ultimately, the exact neuronal event would be extracted from the hemodynamic response based on a “hemodynamic equation” which describes the neurovascular coupling mechanisms. For this, predictability (linearity) of the BOLD signal to neuronal activity is critical. Recent work based on SE BOLD suggests that the microvasculature can behave highly linear to changing neuronal activity [15]. Given that the early part of the GE HRF (in deep gray matter) closely matches that of the SE HRF, we expect a similar linear behavior during the early phase of GE BOLD signals. This opens the possibility to examine the role, in terms of predictability to a neuronal event, of each vessel group in the gray matter separately. Important for such investigations is the accurate tracking of the temporal evolution of BOLD changes in all vessel sizes upon short neuronal activation. This requires however that the estimated hemodynamic responses approximate an impulse response function with high fidelity.

Our results indicate at high field that the early phase of the GE BOLD response, i.e. almost up to the peak, is dominated by microvascular weighted dynamics and hence that it is less affected by the non-specific larger vasculature. It is known that the spatial point spread function of GE BOLD is larger than SE BOLD because of macrovascular contributions and may impede robust analysis of cortical layers and columns [40]. However, we find that these contributions are observable at a later stage of the hemodynamic response and are predominant in the surface gray matter. A fast GE BOLD imaging technique should be able to accurately sample the rising slope of the response allowing for distinguishing, and possibly excluding, the more macrovascular contributions. More studies would be needed to assess the generality of this finding to different cortices, different task designs, or lower resolution acquisitions.

## Conclusion

For systematic analysis of brain function on the cortical laminar/column scale accurate and robust measurements are needed of the microvascular dynamics. Studies traditionally use the GE BOLD contrast because of its superior sensitivity. Here we approximate the microvascular response using the SE BOLD contrast as reference and provide indications that the conventional GE BOLD contrast is weighted towards microvasculature, notably from onset up to the peak of the response in deep gray matter.
